# Interprofessional education through case conferences: Enhancing collaborative skills in psychiatric discharge planning

**DOI:** 10.1002/pcn5.70212

**Published:** 2025-09-30

**Authors:** Yasuhisa Nakamura, Kazuko Ando, Kyoko Otani, Ayako Furuzawa, Mayumi Yoshikawa, Masato Hatsuda, Chizuru Tsubonouchi, Rie Tachi

**Affiliations:** ^1^ Course of Occupational Therapy, Department of Rehabilitation Faculty of Health Sciences Nihon Fukushi University Handa Japan; ^2^ Student Advising and Counseling Office Hitotsubashi University Kunitachi Japan; ^3^ Faculty of Social Welfare Nihon Fukushi University Mihama Japan; ^4^ Department of Nursing Faculty of Nursing Nihon Fukushi University Tokai Japan

**Keywords:** interprofessional education, mental health social worker students, nursing students, occupational therapy student, psychiatric discharge planning

## Abstract

**Aim:**

To examine within‐group pre–post changes associated with a structured interprofessional education (IPE) intervention among occupational therapy (OT), mental health social work (MHSW), and nursing (NS) students in Japan, with a focus on psychiatric discharge planning for long‐term inpatients with schizophrenia.

**Methods:**

We used a one‐group pretest–posttest (quasi‐experimental) design. Senior‐year OT, MHSW, and NS students formed interprofessional teams to develop discharge support plans for a simulated long‐term psychiatric inpatient. The Readiness for Interprofessional Learning Scale (RIPLS; *n* = 194) and the Interdisciplinary Education Perception Scale (IEPS; *n* = 134) were administered immediately before and after the case conference. Linear mixed‐effects models estimated time (pre, post), profession (OT, MHSW, and NS), and time × profession effects with a random intercept for participant. As sensitivity analyses, we conducted repeated‐measures anova on complete cases and ancova adjusting for baseline scores.

**Results:**

RIPLS and IEPS scores increased significantly from pre to post across professions, indicating within‐group improvements in interprofessional readiness and collaborative perceptions. On RIPLS, gains were largest among OT students (small time × profession effect), whereas on IEPS, the time × profession interaction was not significant, and profession differences attenuated after baseline adjustment in ANCOVA. Qualitative feedback suggested a clearer understanding of each profession's role in discharge planning.

**Conclusion:**

This study provides preliminary evidence suggesting the potential value of a structured IPE program for enhancing interprofessional attitudes and competencies in the context of psychiatric discharge planning. Given the one‐group pre–post design, causal inference is limited; controlled, multi‐institutional, and longitudinal studies are needed to validate and extend these findings.

## INTRODUCTION

Interprofessional education (IPE) is increasingly recognized as a critical component in preparing health and medical welfare professionals to collaborate effectively in complex healthcare environments.^1^, ^2^ IPE offers structured opportunities for students from diverse professional backgrounds to learn with, from, and about each other, fostering mutual understanding and ultimately improving the quality of collaborative care.^3^

Previous research has demonstrated that IPE can cultivate more positive attitudes toward interprofessional work (IPW) and enhance teamwork skills among students.^4^, ^5^, ^6^ A growing body of systematic reviews and meta‐analyses supports these findings, indicating that structured IPE interventions significantly improve students' interprofessional attitudes, knowledge, and Interprofessional collaborative competencies.^7^, ^8^, ^9^ In particular, team‐based learning, problem‐based learning, and case‐based conferences have shown promise in enhancing interprofessional readiness and mutual role understanding.^10^, ^11^, ^12^

However, most existing IPE programs focus on acute care or general medical education and overlook the complexities of psychiatric discharge planning.^13^ Internationally, psychiatric IPE has been reported only rarely; for example, Marcussen et al. implemented and evaluated mental health IPE curricula in Denmark and observed preliminary evidence for learners' interprofessional attitudes and collaboration.^14^, ^15^ While not discharge‐specific, these studies help position our work in the global literature and underscore the scarcity of psychiatric IPE research.

Interprofessional collaboration is a critical factor in ensuring continuity of care, particularly in mental health settings where complex psychosocial needs intersect with medical treatment. In psychiatric care, the process of discharge planning often requires coordination among multiple professionals, including physicians, nurses, occupational therapists, and social workers, to address patients' medical, psychological, and social needs. However, despite the importance of discharge planning, opportunities for students in health professions to engage in IPE on this topic remain limited. Addressing this overlooked area through targeted IPE programs can help equip future professionals with the nuanced understanding and shared decision‐making skills required in psychiatric discharge planning.

In Japan, several structural barriers make psychiatric discharge planning particularly challenging. Empirical reports describe persistent difficulties in securing stable housing after long‐term hospitalization, limited availability of community‐based services, and fragmented coordination across medical, welfare, and housing systems.^16^, ^17^ Family members often provide substantial informal care yet report insufficient formal support, which can delay discharge and hinder community reintegration.^18^ In addition, patients and families face challenges in shared decision‐making and care coordination during transitions, indicating a need for explicit training in team‐based, patient‐centered discharge planning.^19^, ^20^

Despite the evident need for IPE in mental health contexts, few programs have directly addressed the skills and attitudes required for effective discharge planning in psychiatric care. Therefore, the present study aimed to assess pre–post changes in students' attitudes and collaborative competencies associated with an IPE intervention involving occupational therapy (OT), mental health worker, and nursing (NS) students. In this program, participants collaborated in mixed‐discipline groups to develop discharge support care plans for long‐term psychiatric inpatients with schizophrenia. By assessing changes in students' attitudes and Interprofessional collaborative competencies, this study seeks to contribute to the optimization of IPE interventions tailored for psychiatric settings.

## METHODS

### Study context

This study was conducted at a large Japanese university that offers professional education programs in OT, mental health social work (MHSW), and NS, each located on separate campuses. The OT program emphasizes improving functional abilities through therapeutic activities; the MHSW program focuses on social support systems and policies for individuals with mental health issues; and the NS program prioritizes health promotion and quality of life through comprehensive care assessments. The geographic separation of these programs supports distinct educational environments aligned with the objectives of each discipline, which may shape students' perspectives in interprofessional activities.

The IPE program evaluated in this study was implemented as part of the formal undergraduate curriculum. Participation was required for enrolled students, and completion was associated with academic credit. This curricular positioning ensured that all students engaged with the program as an integral component of their professional training. The university was therefore chosen as the research site because its specialized programs and structured curriculum provided a robust foundation for IPE aimed at enhancing collaborative competencies in psychiatric discharge planning.

### Study design

**Figure 1:**
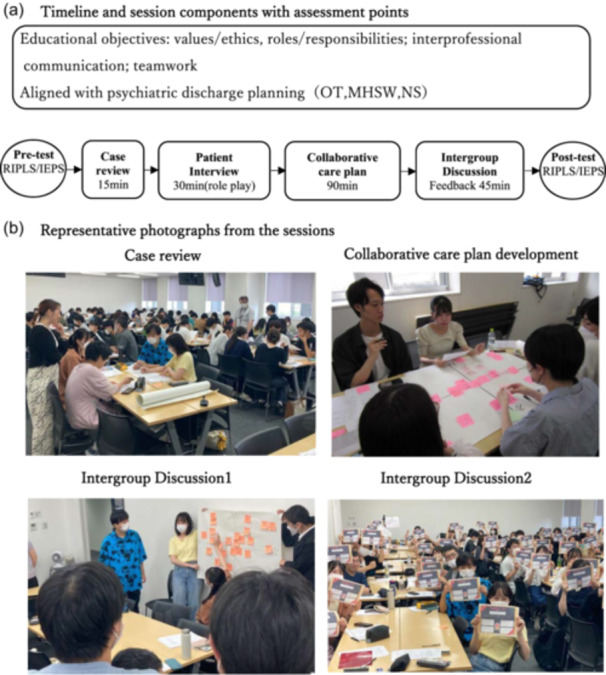
Program objectives and timeline of the interprofessional case conference. (a) Timeline and session components with explicit assessment points: pretest (Readiness for Interprofessional Learning Scale [RIPLS], Interdisciplinary Education Perception Scale [IEPS]) → case review (15 min) → patient interview (30 min) → collaborative care plan development and presentation (90 min) → intergroup discussion/feedback (45 min) → posttest (RIPLS, IEPS). Each group included at least one occupational therapy (OT), one mental health social work (MHSW), and one nursing (NS) student. (b) Representative photographs from the sessions (illustrative; not all stages are shown).

We used a one‐group pretest–posttest (quasi‐experimental) design without a control group; therefore, causal inference is limited. Analyses focus on within‐participant changes from pre to post. Participants completed the Readiness for Interprofessional Learning Scale (RIPLS) and Interdisciplinary Education Perception Scale (IEPS) immediately before and after a structured interprofessional case conference (see Figure 1). The primary outcome was the pre–post change in total scores; secondary outcomes included domain/subscale scores and differences by profession (OT, MHSW, and NS).

### Participants

This study involved senior‐year students enrolled in the OT, MHSW, and NS programs at Nihon Fukushi University during the 2022, 2023, and 2024 academic years. Participation in the IPE program was mandatory for OT and MHSW students as part of their formal curriculum, whereas it was elective for NS students.

Eligibility criteria required students to be in their final year of study, to have completed coursework related to mental health, and to have completed at least 5 days of practical training at a psychiatric hospital or mental health welfare facility.

This study was approved by the Ethics Committee of Nihon Fukushi University (Approval No. 22‐003‐01). All participants received written and verbal explanations of the study purpose, and informed consent was obtained before data collection.

### Interprofessional case conference: Program structure

The educational objectives of the case conference were the following: (1) to foster values and ethics for interprofessional collaboration, (2) to enhance recognition of roles and responsibilities among OT, MHSW, and NS students, (3) to improve interprofessional communication skills, and (4) to strengthen teamwork competencies required for psychiatric discharge planning.

As part of the IPE initiative, small interdisciplinary groups of five students—including at least one student from each of the OT, MHSW, and NS programs—were organized to collaboratively develop care plans for a simulated hospitalized patient with schizophrenia and diabetes (Figure 1). The fictitious case involved a 35‐year‐old woman experiencing repeated hospitalizations, low motivation, poor health management, and challenges with social reintegration.

The program followed a structured, in‐person format at the Tokai Campus of Nihon Fukushi University. Students first spent 15 min reviewing detailed case information and patient history. This was followed by a 30‐min role‐play interview with an instructor acting as the patient to enhance realism and engagement. Next, groups engaged in a 90‐min collaborative session to develop and present discharge care plans, after which a 45‐min group discussion was held to exchange feedback and perspectives.

The teaching team, comprising instructors from all three professions, rotated among groups to facilitate discussions and provide guidance as needed. This structure ensured interdisciplinary collaboration and the integration of medical, social, and NS perspectives into the care planning process.

To provide clarity, Figure 1 illustrates the timeline of the intervention, session components, and the assessment points (administration of RIPLS and IEPS before and after the program).

### RILS and IEPS

The RIPLS and IEPS were used to assess quantitative changes in perceptions and attitudes toward IPE and readiness for interprofessional collaboration. Participants completed both questionnaires before and immediately after the case study sessions.

The validity of the Japanese version of the RIPLS has been confirmed by Tamura et al.^21^ The RIPLS consists of 19 items rated on a 5‐point Likert scale (1 = “strongly disagree” to 5 = “strongly agree”) and is divided into three subscales: teamwork and collaboration (Items 1–9 and 13–16), uniqueness of the profession (Items 12 and 17–19), and IPE opportunities (Items 10–11). Total scores range from 19 to 95, with higher scores indicating a greater readiness for IPE.

*IEPS*. The IEPS, developed by Luecht et al.,^22^ measures changes in students' attitudes following interprofessional learning experiences. This 18‐item instrument uses a 6‐point Likert scale and includes four subscales: competence and autonomy (Items 1, 3, 4, 5, 7, 9, 10, and 13), perceived need for cooperation (Items 6 and 8), perception of actual cooperation (Items 2 and 14–17), and understanding of other values (Items 11, 12, and 18). Total scores range from 18 to 108, with higher scores reflecting stronger perceptions of interprofessional collaboration. Japanese translation and adaptation. Because a fully peer‐reviewed, validated Japanese IEPS was not available, we used a Japanese translation of the original instrument and followed standard cross‐cultural adaptation procedures (two independent forward translations, expert reconciliation, independent back‐translation, and cognitive debriefing with students). The final Japanese wording and the mapping to the four subscales are provided in Supporting Information S1: Table 1; scoring followed the original instrument. To reflect the local context, we also cite a Japanese conference abstract reporting the development and preliminary reliability/validity of a Japanese IEPS.^23^

### Statistical analysis

A linear mixed‐effects model (LMM) was used to evaluate the effects of the interprofessional case conference. Time (pre‐intervention vs. post‐intervention) and profession (OT, MHSW, and NS) were specified as fixed effects; interindividual differences were modeled as a random intercept for participant. The time × profession interaction was included to examine whether changes over time differed by profession. Models were estimated by restricted maximum likelihood (REML), and denominator degrees of freedom were obtained using the Satterthwaite approximation. LMMs were chosen a priori to accommodate unbalanced group sizes, model participant‐level heterogeneity, and—given only two time points—use all available observations under a missing‐at‐random assumption without restricting analyses to complete cases. With two occasions, we specified a random‐intercept structure, as alternative residual covariance structures (e.g., AR[1]) are not identifiable in this setting.

The dependent variables were the total scores and subscales of the RIPLS and IEPS. The RIPLS subscales were teamwork, IPE, and professional identity; the IEPS subscales assessed competence and autonomy, recognition of the need for collaboration, perception of actual collaboration, and understanding of others' values. Type III tests of fixed effects were used for time, profession, and their interaction.

When a significant interaction was identified, simple main‐effects analyses were conducted to evaluate within‐profession change over time and between‐profession differences at each time point. Multiple comparisons were adjusted using the Bonferroni method. As sensitivity analyses, we conducted (a) repeated‐measures anova on complete cases (factors: time and profession) and (b) ancova with posttest scores as the dependent variable, pretest scores as a covariate, and profession as a fixed factor. Both analyses reproduced the LMM inferential pattern (Supporting Information S2: Table 2). For transparency, we report estimated marginal means, mean changes with 95% confidence intervals (CIs), and *p*‐values for all primary outcomes. A two‐sided significance level of *p* < 0.05 was used. Analyses were performed in IBM spss Statistics, version 27.0 (IBM).

*Qualitative analysis*. We adopted a conventional content analysis approach. All free‐text responses describing the characteristics of other professions and learning outcomes from developing a discharge support care plan were transcribed verbatim and subjected to open coding. Two researchers independently generated initial codes, compared them, and resolved discrepancies through discussion until consensus was reached. Codes were then organized into subcategories and categories to capture recurring themes related to interprofessional learning. Representative quotations were identified to illustrate each category. An audit trail and analytic memos were maintained to enhance the dependability of the analysis.

## RESULTS

### Participant demographics

A total of 200 students were initially enrolled in the IPE program: 105 OT students, 79 MHSW students, and 16 NS students. After excluding students who declined to participate (2 OT, 3 MHSW, and 1 NS), 194 students were included in the final analysis: 103 OT (28 males, 75 females), 76 MHSW (20 males, 56 females), and 15 NS (4 males, 11 females). The relatively small number of NS students reflected differences in academic calendars and mandatory clinical rotations, which limited their availability for participation.

All 194 participants completed the RIPLS questionnaire. Responses to the IEPS questionnaire were obtained from 70 participants in the OT group (23 males, 47 females; mean age 21.3 ± 0.9 years), 49 in the MHSW group (17 males, 32 females; mean age 21.6 ± 1.0 years), and 15 in the NS group (4 males, 11 females; mean age 21.4 ± 1.3 years), yielding a total of 44 males and 90 females across the IEPS sample. Because the IEPS was introduced starting in the second year of the study, data were collected only from participants who took part from Year 2 onward.

Participant numbers for enrollment, final analysis, and questionnaire completion are summarized in Table 1.

**Table 1:** Participant demographics and questionnaire completion.

Program	Enrolled	Final analyzed	RIPLS completed, *n*	IEPS completed, *n*
OT	105	103	103	70
MHSW	79	76	76	49
NS	16	15	15	15
Total	200	194	194	134

*Note*: IEPS was introduced starting in the second year of the study; therefore, IEPS data were collected only from those participants.

Abbreviations: IEPS, Interdisciplinary Education Perception Scale; MHSW, mental health social work; NS, nursing; OT, occupational therapy; RIPLS, Readiness for Interprofessional Learning Scale.

### Main statistical findings

Sensitivity analyses (RM‐anova, ancova) reproduced the LMM inferential pattern (Supporting Information S2: Table 2). For RIPLS, the time effect was significant, and the time × profession interaction was small but significant. For IEPS, the time effect was significant, whereas the interaction was not; the omnibus profession effect attenuated after baseline adjustment (*p* = 0.068).

### Main effect of time

Table 2 presents the main effects of time on RIPLS and IEPS.

1. Items related to RIPLS
  A significant main effect of time was observed for the total RIPLS score (*F*(1, 191) = 39.735, *p* < 0.001), with significantly improved scores after the intervention. The RIPLS teamwork and collaboration scores demonstrated a significant main effect of time (*F*(1, 191) = 31.804, *p* < 0.001), indicating improvement following the intervention. The RIPLS uniqueness of profession score also showed a significant main effect of time (*F*(1, 191) = 4.231, *p* = 0.041), reflecting post‐intervention improvement. Similarly, the RIPLS learning opportunities score revealed a significant main effect of time (*F*(1, 191) = 5.904, *p* = 0.016), confirming the enhanced scores after the intervention.
2. Items related to IEPS
  **Table 2:** Analysis of main and interaction effects of time and profession on Readiness for Interprofessional Learning Scale (RIPLS) and Interdisciplinary Education Perception Scale (IEPS) scores.

Measurement item	Main effect of time *F* (*p*)	Main effect of profession *F* (*p*)	Time × profession interaction *F* (*p*)
RIPLS			
Total score	*F*(1, 191) ＝39.735	*F*(2, 191) ＝1.360	*F*(2, 191) ＝3.316
	*p*＝0.001	*p*＝0.259	*p*＝0.038
Teamwork and collaboration	*F*(1, 191) ＝31.804	*F*(2, 191) ＝4.963	*F*(2, 191) ＝3.770
	*p*＝0.001	*p*＝0.008	*p*＝0.025
Uniqueness of profession	*F*(1, 191) ＝4.231	*F*(2, 191) ＝2.474	*F*(2, 191) ＝0.531
	*p*＝0.041	*p*＝0.087	*p*＝0.589
Learning opportunities	*F*(1, 191) ＝5.904	*F*(2, 191) ＝0.678	*F*(2, 191) ＝1.155
	*p*＝0.016	*p*＝0.509	*p*＝0.317
IEPS			
Total score	*F*(1, 131) ＝35.119	*F*(2, 131) ＝4.566	*F*(2, 131) ＝2.034
	*p* < 0.001	*p*＝0.012	*p*＝0.135
Competence and autonomy	*F*(1, 131) ＝35.955	*F*(2, 131) ＝5.730	*F*(2, 131) ＝1.835
	*p* < 0.001	*p*＝0.004	*p*＝0.164
Perceived need for cooperation	*F*(1, 131) ＝2.690	*F*(2, 131) ＝5.230	*F*(2, 131) ＝0.149
	*p*＝0.103	*p*＝0.007	*p*＝0.862
Perception of actual cooperation	*F*(1, 131) ＝33.460	*F*(2, 131) ＝2.137	*F*(2, 131) ＝3.591
	*p* < 0.001	*p*＝0.122	*p*＝0.030
Understanding others' value	*F*(1, 131) ＝7.595	*F*(2, 131) ＝3.387	*F*(2, 131) ＝0.206
	*p*＝0.007	*p*＝0.037	*p*＝0.814

*Note*: Values are presented as mean ± standard error. Group differences were assessed using linear mixed models with Bonferroni correction. *p* < 0.05 was considered statistically significant.
  A significant main effect of time was observed for the total IEPS score (*F*(1, 131) = 35.119, *p* < 0.001), with scores increasing significantly after the intervention. The IEPS competence and autonomy scores also demonstrated a significant main effect of time (*F*(1, 131) = 35.955, *p* < 0.001), with post‐intervention scores showing a marked increase. In contrast, the IEPS Perceived need for cooperation score did not show a significant main effect of time (*F*(1, 131) = 2.034, *p* = 0.135). Similarly, the IEPS understanding others' values score revealed a significant main effect of time (*F*(1, 131) = 7.595, *p* = 0.007), confirming improved post‐intervention scores.

### Main effect of profession

Table 2 presents the main effects of professional student groups on the RIPLS and IEPS.

1. Items related to RIPLS
  A significant main effect of professional group was observed for RIPLS teamwork and collaboration scores, *F*(2, 191) = 4.963, *p* = 0.008. The OT group demonstrated significantly higher scores than the MHSW and NS groups.
2. Items related to IEPS
  A significant main effect of professional group was observed for the IEPS total score (F(2,131) = 4.566, *p* = 0.012), with the OT group showing higher scores than students from other professional groups. Specifically, significant differences were observed in the subscales of competence and autonomy (*F*(2, 131) = 5.730, *p* = 0.004), Perceived need for cooperation (*F*(2, 131) = 5.230, *p* = 0.007), and understanding others' values (*F*(2, 131) = 3.387, *p* = 0.037), with the OT group exhibiting significantly higher scores.

### Interaction between profession and time

Table 2 highlights the interaction effects between time and professional group, and Table 3 details the pre‐ and post‐intervention scores and score changes for each professional group.

1. RIPLS total score
  A significant interaction effect between time and profession was identified for the RIPLS total score (*F*(2,191) = 3.316, *p* = 0.038), with the OT group demonstrating the largest improvement following the intervention. The OT group achieved a pre‐intervention score of 76.03 ± 0.78 and a post‐intervention score of 80.15 ± 0.78, reflecting a score change of 4.12 ± 0.78. In comparison, the MHSW group showed a score change of 3.05 ± 0.82, while the NS group demonstrated a change of 2.00 ± 1.84. Post‐intervention, the OT group's scores were significantly higher than those of the MHSW (*p* = 0.021) and NS groups (*p* = 0.008).
2. RIPLS teamwork and collaboration score
  A significant interaction effect between time and profession was also observed for the RIPLS teamwork and collaboration scores (*F*(2, 191) = 3.770, *p* = 0.025). The OT group recorded a pre‐intervention score of 54.91 ± 0.62 and a post‐intervention score of 59.69 ± 0.62, resulting in a score change of 4.78 ± 0.62. In contrast, the MHSW group exhibited a score change of 2.91 ± 0.64, while the NS group showed a score change of 1.33 ± 1.43. Post‐intervention, the OT group's scores were significantly higher than those of the MHSW (*p* = 0.012) and NS groups (*p* = 0.003).
3. IEPS perception of actual cooperation score
  **Table 3:** Changes in Readiness for Interprofessional Learning Scale (RIPLS) and Interdisciplinary Education Perception Scale (IEPS) scores by profession: pre‐ and post‐intervention analysis with group comparisons.

Measurement item	Profession	Pre‐intervention score	Post‐intervention score	Score change	Group comparison (Bonferroni correction)
RIPLS total score	OT	76.03 ± 0.78	80.15 ± 0.78	4.12 ± 0.78	OT > SW (*p* = 0.021), OT > NS (*p* = 0.008)
	MHSW	77.09 ± 0.82	80.14 ± 0.82	3.05 ± 0.82	
	NS	78.33 ± 1.84	80.33 ± 1.84	2.00 ± 1.84	
RIPLS teamwork and collaboration	OT	54.91 ± 0.62	59.69 ± 0.62	4.78 ± 0.62	OT > SW (*p* = 0.012), OT > NS (*p* = 0.003)
	MHSW	54.80 ± 0.64	57.71 ± 0.64	+2.91 ± 0.64	
	NS	57.23 ± 1.43	58.56 ± 1.43	1.33 ± 1.43	
IEPS perception of actual cooperation	OT	24.12 ± 0.35	25.81 ± 0.35	1.69 ± 0.35	OT > SW (*p* = 0.045)
	MHSW	24.49 ± 0.42	25.49 ± 0.42	1.00 ± 0.42	
	NS	26.07 ± 0.76	27.07 ± 0.76	1.00 ± 0.76	

*Note*: Values are presented as mean ± standard error. “Score change” indicates the difference between post‐ and pre‐intervention scores. Group comparisons were performed using linear mixed models with Bonferroni correction. *p* < 0.05 was considered statistically significant.

Abbreviations: MHSW, mental health social work students; NS, nursing students; OT, occupational therapy students.
  A significant interaction effect between time and profession was also found for the IEPS perception of actual cooperation scores (*F*(2,131) = 3.591, *p* = 0.030). The OT group achieved a pre‐intervention score of 24.12 ± 0.35 and a post‐intervention score of 25.81 ± 0.35, corresponding to a score change of 1.69 ± 0.35. In contrast, both the MHSW and NS groups exhibited a score change of 1.00 ± 0.42 and 1.00 ± 0.76, respectively. The post‐intervention score of the OT group was significantly higher than that of the MHSW group (*p* = 0.045).

RIPLS total:

Post hoc pairwise comparisons revealed that OT students scored significantly higher than did MHSW students (*p* = 0.007), with a mean difference of 2.586 (95% CI [0.562, 4.609]). No significant differences were observed between the OT and NS students (*p* = 1.000) or between the MHSW and NS (*p* = 0.366).

RIPLS teamwork:

For RIPLS teamwork, OT students scored significantly higher than MHSW students (*p* = 0.007), with a mean difference of 2.586 (95% CI [0.562, 4.609]). There were no significant differences between the OT and NS (*p* = 1.000) or between the MHSW and NS (*p* = 0.366).

RIPLS professionalism:

The comparisons of RIPLS professionalism did not reveal any significant differences between professions. The mean differences were 0.569 (95% CI [−1.253, 0.116], *p* = 0.138) for OT versus SW, 0.241 (95% CI [−1.009, 1.492], *p* = 1.000) for OT versus NS, and −0.810 (95% CI [−2.089, 0.468], *p* = 0.383) for SW versus NS.

RIPLS IPE:

Similarly, no significant differences were observed in RIPLS IPE subscale scores. The mean differences were 0.248 (95% CI [−0.829, 0.332], *p* = 0.909) for OT versus SW, 0.125 (95% CI [−0.936, 1.185], *p* = 1.000) for OT versus NS, and −0.373 (95% CI [−1.457, 0.712], *p* = 1.000) for SW versus NS.

### Interprofessional learning through discharge support care plan development

1. Interprofessional learning through discharge support care plan development:
  Understanding of OT by other professionals
  Comments from MHSW
  Occupational therapists assess functional abilities and daily living skills by focusing on functional improvements and recovery. Training and activities tailored to the patients' interests were utilized to facilitate discharge planning.
  Comments from NSs
  Occupational therapists evaluate patients' abilities and limitations in activities of daily living (ADL) and instrumental activities of daily living (IADL) and implement appropriate interventions accordingly.
  Interprofessional learning outcomes
  Through IPE, students from other disciplines learned that occupational therapists should integrate medical and welfare perspectives, conduct detailed ADL and IADL assessments, develop individualized programs, and implement training based on these assessments.
2. Interprofessional learning through discharge support care plan development: understanding of MHSW by other professionals
  Comments from OT
  Mental health social workers focus on patients' needs and consider environmental support using social systems to ensure the continuity of life after discharge.
  Comments from NSs
  Discharge support extends beyond the discharge process itself, encompassing continued post‐discharge life. Mental health social workers consider the use of employment and housing support systems as well as informal support from family and friends.
  Interprofessional learning outcomes
  Through the IPE, students from other disciplines recognized that mental health social workers view discharge support as a comprehensive process beyond medical care, aiming to enable patients to sustain independent living in the community.
3. Interprofessional learning through discharge support care plan development: understanding of NSs by other professionals
  Comments from OT
  Nurses possess extensive knowledge of diseases and pharmacotherapy, and emphasize support for medication adherence, nutrition, and financial management to facilitate patients' health maintenance.

Comments from MHSW

Nurses primarily provide support focusing on symptom management, patient insights into their condition, and medical supervision.

Interprofessional learning outcomes

Through IPE, students from other disciplines understood that nurses prioritized enabling patients to manage their health post‐discharge, emphasizing patient education for disease awareness and lifestyle improvements.

## DISCUSSION

This study evaluated the impact of an IPE program wherein OT, MHSW, and NS students collaboratively developed discharge care plans for patients with schizophrenia. The case conference format significantly improved RIPLS and IEPS scores across all student groups, suggesting enhanced interprofessional readiness and Interprofessional collaborative competencies. These findings align with previous studies indicating that structured IPE interventions enhance students' interprofessional attitudes, knowledge, and teamwork skills.^7^, ^8^ Furthermore, the qualitative data enriched our understanding by highlighting students' internalized insights regarding the roles of different professions in psychiatric discharge planning.^24^

Notably, the magnitude of improvement varied among professional groups. OT students demonstrated the largest gains, particularly in teamwork‐related competencies. For these students, the IPE experience was especially meaningful as it integrated psychosocial considerations into their traditional emphasis on functional recovery. Qualitative responses revealed that many OT students developed a deeper understanding of how medical and social factors must be coordinated in effective discharge planning—an insight that was less apparent before the intervention.

One plausible explanation is that OT students in Japan are often placed individually in clinical training settings, which provide limited opportunities for interprofessional dialogue.^25^, ^26^ In contrast, NS and social work students more frequently encounter interprofessional teams during their training. Accordingly, the structured IPE case conference may have offered OT students a particularly valuable opportunity to engage in interprofessional communication and teamwork, resulting in greater improvement.

Several OT students reported realizing the importance of collaborating with social workers and nurses to ensure a smooth transition from hospital to community living. For example, one OT student commented, “I used to focus mainly on improving patients' daily activities, but now I understand that working together with other professionals is essential for supporting patients' lives after discharge.”

These results echo previous research, which highlights the value of realistic, clinically grounded IPE in fostering meaningful interdisciplinary learning.^27^, ^28^

MHSW students also reported substantial preliminary evidence. While many initially approached discharge planning primarily from a social welfare perspective, participation in the IPE program led them to a deeper appreciation of occupational therapists' roles in supporting patients' functional recovery, particularly in relation to ADL and IADL. Several MHSW students noted that their understanding of rehabilitation expanded to include not only social support but also the importance of fostering patients' independence through collaborative care. For example, one student remarked, “I realized that occupational therapists play a key role in helping patients regain their daily life skills, which is essential for successful community reintegration after discharge.” This shift in perspective aligns with earlier research confirming that IPE interventions can reshape participants' understanding of other professions and enhance collaborative practice.^7^, ^8^

Internationally, related psychiatric IPE programs have been reported; however, these initiatives have generally focused on inpatient mental health education more broadly rather than discharge‐specific competencies.^14^, ^15^ Building on this background, our study contributes by explicitly targeting psychiatric discharge planning, integrating psychosocial, functional, and community perspectives within a realistic case conference format.

In contrast, NS students exhibited smaller quantitative improvements, likely due to their already extensive clinical exposure and relatively higher baseline scores. However, qualitative feedback revealed that the IPE program helped NS students gain a deeper understanding and appreciation of the unique contributions of rehabilitation and social welfare professionals to patient care. Many NS students noted that they became more aware of how occupational therapists and social workers support patients' transitions from hospital to community, and recognized the importance of interdisciplinary collaboration beyond routine clinical duties. For instance, one student reflected, “I realized that effective discharge planning requires us to communicate closely with OT and social workers to ensure patients receive comprehensive support after leaving the hospital.” These qualitative insights highlight the added educational value of interdisciplinary collaboration, even for professions that are already accustomed to working in teams.

This study contributes importantly to the under‐explored area of psychiatric discharge planning, which demands integrated psychosocial, functional, and community perspectives.^16^ Unlike general medical or acute care settings, psychiatric discharge necessitates close interprofessional collaboration, particularly amidst global shifts toward deinstitutionalization and community‐oriented mental healthcare.^16^ Our findings strongly support the development of targeted, realistic IPE interventions that reflect these clinical realities and complexities.

The observed interaction between profession and intervention within‐group improvements suggests tailored learning impacts depending on curricular alignment. The discharge planning focus particularly suited OT curricula, providing concrete opportunities to apply and enhance their specific expertise. However, valuable insights were also evident for MHSW and NS students, underscoring the program's broader educational within‐group improvements. Future IPE initiatives should explicitly consider curricular integration and professional specificity to optimize interdisciplinary outcomes across diverse educational contexts.

Several limitations must be carefully considered. First, the small sample size of NS students limited statistical power; thus, results related to this group must be interpreted cautiously. Second, the single‐institution setting and lack of longitudinal follow‐up constrain the generalizability and sustainability of these findings. Third, the IEPS questionnaire was introduced only from the second year onward, resulting in a smaller sample size for related analyses and limiting the ability to directly compare changes across all participant groups throughout the entire study period. Fourth, the absence of a control group means that observed improvements could reflect factors other than the IPE program itself, such as maturation or testing effects, and it is possible that similar gains might occur through less resource‐intensive activities. Finally, qualitative insights, although valuable, may have been subject to researcher bias or missed nuances due to the absence of more rigorous analytical procedures such as systematic thematic coding or multiple independent raters. Future research should address these limitations by incorporating larger, balanced samples from multiple institutions, integrating longitudinal assessments, and applying more structured qualitative analyses.

Looking ahead, future research should include larger and more balanced samples across multiple institutions and expand participation to additional health professions, such as clinical psychology, pharmacy, and physical therapy.^20^ The adoption of longitudinal study designs and more rigorous qualitative methods will allow a better understanding of the sustained impact of IPE. Furthermore, investigating interprofessional interventions in diverse clinical and educational settings—including the use of real patient cases, immersive clinical training, and robust qualitative methodologies—will help refine educational frameworks and further strengthen collaborative practice in healthcare.^29^ Such ongoing efforts will be essential for developing robust and widely applicable educational models for interprofessional practice, ultimately improving patient outcomes and the quality of healthcare teamwork.

## CONCLUSION

This study provides preliminary evidence supporting the potential value of a structured IPE program focused on psychiatric discharge planning, suggesting potential benefits in enhancing students' interprofessional collaborative competencies and professional awareness. By involving OT, MHSW, and NS students in realistic, case‐based, team‐based activities, the program offered valuable experiential learning closely aligned with real‐world clinical coordination. These findings should be interpreted with caution, as they suggest possible benefits rather than definitive effects, and they underscore the importance of IPE initiatives that address the inherent complexities of mental healthcare, particularly discharge processes requiring integration of medical, psychosocial, and community‐based support systems. Future research should include balanced cohorts across professions, address institutional and curricular barriers, and pursue multi‐institutional collaborations. Such efforts will facilitate the development of robust and broadly applicable educational models for interprofessional practice, effectively responding to contemporary healthcare demands.

## AUTHOR CONTRIBUTIONS

**Yasuhisa Nakamura**: Conceptualization; methodology; investigation; writing—original draft; writing—review and editing; corresponding author; funding acquisition. **Kazuko Ando**: Conceptualization; methodology; investigation; data curation; writing—review and editing; supervision; funding acquisition. **Kyoko Otani**: Conceptualization; methodology; investigation; data curation; writing—review and editing; project administration; supervision; funding acquisition. **Ayako Furuzawa**: Investigation; data curation; writing—review and editing; project administration; supervision; funding acquisition. **Mayumi Yoshikawa**: Investigation; data curation; funding acquisition. **Masato Hatsuda**: Investigation; data curation. **Chizuru Tsubonouchi**: Investigation; data curation. **Rie Tachi**: Investigation; data curation.

## CONFLICT OF INTEREST STATEMENT

The authors declare no conflicts of interest.

## ETHICS APPROVAL STATEMENT

The study protocol was approved by the Clinical Research Ethics Committee of Nihon Fukushi University, Japan (Approval No. 22‐003‐01).

## PARTICIPANT CONSENT STATEMENT

All participants provided informed consent to take part in the study and for the publication of anonymized findings. Participation was voluntary and confidentiality was ensured.

## PATIENT CONSENT STATEMENT

N/A.

## CLINICAL TRIAL REGISTRATION

N/A.

## Supporting information

Supporting Information.

Supporting Information.

## Data Availability

The data that support the findings of this study are available from the corresponding author upon reasonable request.
