# Supplementary material for: Interprofessional education through case conferences: Enhancing collaborative skills in psychiatric discharge planning
Source: PCN Rep. 2025 Sep 30;4(4):e70212. doi: 10.1002/pcn5.70212 (PMC12481829; doi:10.1002/pcn5.70212)
Supplement: Supplementary file 1 — Supporting Information. [file PCN5-4-e70212-s002.docx]

Supplementary Table S1. Japanese wording of the Interdisciplinary Education Perception Scale (IEPS) and subscale assignment

| Item | Subscale | Japanese wording |
| --- | --- | --- |
| １ | Competence and autonomy | 私と同じ専門職種の人々は十分な教育を受けている。 |
| ２ | Perception of actual cooperation | 私と同じ専門職種の人々は、ほかの専門職種の人々と緊密に連携できる。 |
| ３ | Competence and autonomy | 私と同じ専門職種の人々は自律的に行動できる。 |
| ４ | Competence and autonomy | ほかの専門職種の人々は、私の専門職種の業務を尊重している。 |
| ５ | Competence and autonomy | 私と同じ専門職種の人々は、自分達で掲げた目的や目標に対し非常に前向きである。 |
| ６ | Perceived need for cooperation | 私と同じ専門職種の人々は、ほかの専門職と協力すべきである。 |
| ７ | Competence and autonomy | 私と同じ専門職種の人々は、自分達の医療への貢献度や実績に関して自信がある。 |
| ８ | Perceived need for cooperation | 私と同じ専門職種の人々は、ほかの専門職を信頼して業務を任せるべきである。 |
| ９ | Competence and autonomy | ほかの専門職種の人々は、私と同じ専門職種の人々を高く評価している。 |
| 10 | Competence and autonomy | 私と同じ専門職種の人々は専門家としてのお互いの判断を尊重する。 |
| 11 | Understanding of other values | 私と同じ専門職種の人々はほかの専門職よりも地位が高い。 |
| 12 | Understanding of other values | 私と同じ専門職種の人々はほかの専門職の能力や貢献を理解しようと努力する。 |
| 13 | Competence and autonomy | 私と同じ専門職種の人々は非常に能力が高い。 |
| 14 | Perception of actual cooperation | 私と同じ専門職種の人々は積極的にほかの専門職と情報や資源を共有しようとする。 |
| 15 | Perception of actual cooperation | 私と同じ専門職種の人々はほかの専門職とよい関係を築いている。 |
| 16 | Perception of actual cooperation | 私と同じ専門職種の人々はほかの専門職種の人々を高く評価している。 |
| 17 | Perception of actual cooperation | 私と同じ専門職種の人々は専門職内で互いに円滑に協働できる。 |
| 18 | Understanding of other values | ほかの専門職種の人々は頻繁に私と同じ専門職種の人々の助言を求める。 |

Response scale: 6-point Likert (1 = strongly disagree, 6 = strongly agree). Total score 18–108; scoring and subscales identical to Luecht et al. (1990). Translation followed standard cross-cultural adaptation procedures (two forward translations, reconciliation, back-translation, cognitive debriefing).
