# Supplementary material for: Interprofessional education through case conferences: Enhancing collaborative skills in psychiatric discharge planning
Source: PCN Rep. 2025 Sep 30;4(4):e70212. doi: 10.1002/pcn5.70212 (PMC12481829; doi:10.1002/pcn5.70212)
Supplement: Supplementary file 2 — Supporting Information. [file PCN5-4-e70212-s001.docx]

**Supplementary Table S2. Sensitivity analyses for total scores (RIPLS, IEPS)**

| Scale | Analysis | Effect | df | F | p | partial η² |
| --- | --- | --- | --- | --- | --- | --- |
| RIPLS | RM-ANOVA(complete cases) | Time | 1,191 | 39.735 | <.001 | .172 |
|  |  | Time×Profession | 2,191 | 3.316 | .038 | .034 |
|  |  | Profession (between) | 2,191 | 1.360 | .259 | .014 |
| RIPLS | ANCOVA(Post~Pre +Profession) | Pre (covariate) | 1,190 | 145.306 | <.001 | .433 |
|  |  | Profession | 2,190 | 3.721 | .026 | .038 |
| IEPS | RM-ANOVA (complete cases) | Time | 1,131 | 35.119 | <.001 | .211 |
|  |  | Time×Profession | 2,131 | 2.034 | .135 | .030 |
|  |  | Profession (between) | 2,131 | 4.566 | .012 | .065 |
| IEPS | ANCOVA (Post ~ Pre + Profession) | Pre (covariate) | 1,130 | 117.025 | <.001 | .474 |
|  |  | Profession | 2,130 | 2.742 | .068 | .040 |

**Footnote.** RM-ANOVA was conducted on complete cases. ANCOVA modeled post-test scores as the dependent variable, with pre-test scores as a covariate and profession as a fixed factor. All p-values are two-sided (α = .05). Effect sizes are reported as partial η². Because the IEPS was introduced in year 2, complete-case analyses for IEPS were based on n = 134.
